# Analysis of non-invasive gait recording under free-living conditions in patients with Parkinson’s disease: relationship with global cognitive function and motor abnormalities

**DOI:** 10.1186/s12883-020-01729-w

**Published:** 2020-04-29

**Authors:** Hiroo Terashi, Takeshi Taguchi, Yuki Ueta, Yoshihiko Okubo, Hiroshi Mitoma, Hitoshi Aizawa

**Affiliations:** 1 Department of Neurology, Tokyo Medical University, 6-7-1 Nishishinjuku, Shinjuku-ku, Tokyo, 160-0023 Japan; 2 Medical Education Promotion Center, Tokyo Medical University, 6-7-1 Nishishinjuku, Shinjuku-ku, Tokyo, 160-0023 Japan

**Keywords:** Parkinson’s disease, Free-living gait, Wearable sensor, Portable device, Global cognitive function

## Abstract

**Background:**

We investigated the gait characteristics of patients with Parkinson’s disease (PD), under free-living conditions, using a wearable device, and assessed their relationships with global cognitive function and motor abnormalities.

**Methods:**

The study subjects comprised patients with PD aged < 80 years, with a Mini-Mental State Examination (MMSE) score of ≥20, free of any motor complications. A wearable sensor with a built-in tri-axial accelerometer was waist-mounted on each patient, and continuous, 24-h records were obtained. The mean gait cycle duration and mean gait acceleration amplitude, under free-living conditions, were computed and analyzed to determine their relationship with disease duration, MMSE score, Unified Parkinson’s Disease Rating Scale (UPDRS) Part III score, and postural instability and gait difficulty (PIGD) score.

**Results:**

The study included 106 consecutive patients with PD. The mean gait cycle duration was 1.18 ± 0.12 s, which was similar to that of the normal controls. However, the mean gait acceleration amplitude of PD patients (1.83 ± 0.36 m/s^2^) was significantly lower than that of the control (*p* < 0.001). In PD patients, the mean gait acceleration amplitude correlated with the MMSE (*β =* 0.197, *p =* 0.028), UPDRS Part III (*β =* − 0.327, *p* < 0.001), and PIGD (*β =* − 0.235, *p =* 0.008) scores.

**Conclusions:**

The gait rhythm of PD patients is preserved at levels similar to those of normal subjects. However, the mean gait acceleration amplitude was significantly reduced in patients with PD. The results indicate that gait acceleration amplitude correlates with the severity of motor disorders and global cognitive function.

## Background

Parkinson’s disease (PD) is a typical neurodegenerative disease that affects mainly middle-aged and elderly people. In Japan, the prevalence of PD is increasing with the aging population [1]. Patients with PD exhibit characteristic gait disorders related to core motor symptoms, such as rest tremor, muscle rigidity, and bradykinesia. Gait disorders are important features that can directly impair the functional independence and quality of life of patients with PD; however, proper assessment of gait abnormality can be difficult in some patients [2]. In routine clinical practice, PD-related gait disturbance is assessed by visual observation of the gait in the examination room, together with the use of one or more clinical assessment scales, such as the Unified Parkinson’s Disease Rating Scale (UPDRS), the updated version of UPDRS, and Movement Disorder Society-sponsored version of the UPDRS (MDS-UPDRS) [3]. However, no doubt such assessment performed in the confined space of the examination room within a limited test duration has limitations. Based on recent advances in digital medical devices, wearable sensors have been used to provide non-invasive clinical assessment of gait disturbances in patients with PD [4, 5]. The advantages of wearable sensors include the relatively easy and continuous monitoring of the motion states of patients in their daily lives, without any temporal or spatial constraints, and objective assessment of their states [4]. In Japan, a locally-manufactured portable device with built-in tri-axial accelerometer (MIMAMORI-Gait, LSI Medience Corporation, Japan) has been approved for use by the Ministry of Health, Labour and Welfare. It is mainly used to evaluate gait and physical activity in patients with neurodegenerative diseases, such as PD and progressive supranuclear palsy and those with parkinsonism, among other diseases [6–11]. Although the use of wearable sensors provides reliable assessment of the gait of PD patients different from the conventional assessment performed in the examination room or laboratory, their use and assessment of gait parameters remain poorly evaluated.

The aim of this study was to determine the gait characteristics of PD patients under free-living conditions. For this purpose, we measured the mean gait cycle duration and mean gait acceleration amplitude in PD patients using the MIMAMORI-Gait sensor, analyzed the gait characteristics, and determined the relation of such characteristics with global cognitive function and motor symptoms.

## Methods

### Participants

The study subjects were PD patients who visited the Outpatient Clinic of the Department of Neurology, Tokyo Medical University Hospital between July 2009 and August 2017, who fulfilled the following criteria: *1)* age < 80 years, *2)* free of motor complications, such as wearing off or dyskinesia, and *3)* provided written informed consent to participate in this study. Patients with any of the following conditions were excluded from the study: a Mini-Mental State Examination (MMSE) score of < 20, or another concurrent neurodegenerative disease, or current treatment for psychiatric disorder, stroke, arthralgia, or spinal disease complications that affected activities of daily living. PD was diagnosed according to the criteria of the United Kingdom Parkinson’s Disease Society Brain Bank [12]. Brain MRI was performed in all the patients to rule out the presence of other neurodegenerative diseases, stroke, and idiopathic normal pressure hydrocephalus. The study protocol was approved by the Medical Ethics Committee of Tokyo Medical University Hospital. Furthermore, the study also adhered to the tenets of the 1964 Helsinki declarations.

### Clinical assessment

Global cognition was assessed using the MMSE. Motor severity was assessed using the UPDRS Part III scores. In addition, in the assessment with the UPDRS Part III, postural instability and gait disorder (PIGD) scores (the sum of scores on UPDRS “PIGD items” 13–15, 29, and 30) were also assessed separately [13].

### Gait assessment

#### Equipment

**Fig. 1:**
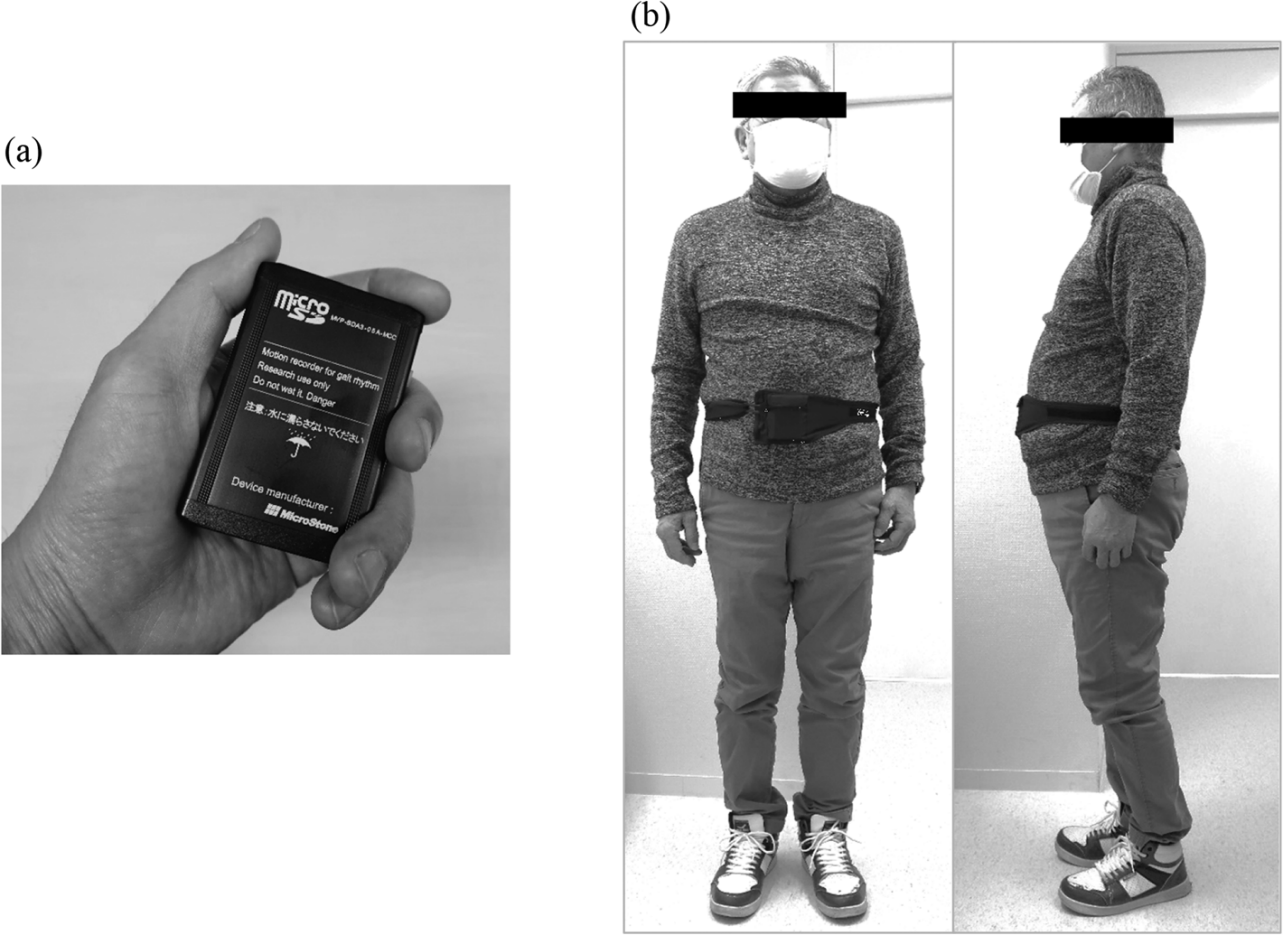
(**a**) The MIMAMORI-Gait device, (**b**) The device attached to the patient

The MIMAMORI-Gait is a small device measuring 8 × 6 × 2 cm (weight, 80 g) that measures, in three dimensions (*a*_x_, *a*_y_, *a*_z_), accelerations caused by limb and trunk movements, and those induced by step-in and kick-off during gait (Fig. 1) [6, 14–16]. The device was secured with a belt to the front and center of the subject’s waist (Fig. 1). When standing in the anatomical position, the orientations of the X, Y, and Z-axes were medial/lateral, vertical, and anterior/posterior, respectively. Positive X values corresponded to leftward acceleration, positive Y values to upward acceleration, and positive Z values to forward acceleration. The subject was instructed to wear the device at all times during a 24-h period, except when changing clothes or taking a shower/bath.

Patients were asked to adhere to their ordinary daily routines and to avoid exceptional physical activities, such as shopping and exercise outside the home. The 24-h period was a continuous recording starting between 10:00 am and 12:00 pm. Gait analysis was also conducted in 15 age- and height-matched normal control subjects (age, 67.9 ± 4.7 years, 9 men and 6 women).

The MIMAMORI-Gait records at a sampling rate of 10 ms. The sensor resolution is approximately 0.16 m/s^2^. Data were automatically stored on a micro-SD card. When recording was completed, the absolute value of the acceleration vectors (*a*; *a*^2^ = *a*_x_^2^ + *a*_y_^2^ + *a*_z_^2^) was automatically computed and displayed graphically on the PC monitor. A fully charged MIMAMORI-Gait can record continuously for 40 h (Additional file 1) [6, 14, 15, 17].

#### Identification of acceleration induced by gait motion

The acceleration vectors caused by stepping can be distinguished from those caused by other limb and trunk movements or by unexpected artifacts, based on the mathematical method of “pattern matching”, as reported previously [6, 14, 15, 17]. First, attention focused on a relatively strong signal region (e.g., *a* > 1 m/s^2^) in the acceleration time series, and a three-dimensional template wave (*a*_x_, *a*_y_, *a*_z_) with a duration of about 0.5 s was arbitrarily chosen. The cross-correlation *CC*(*t*) between this wave and another wave with a time shift *t* chosen from the whole time series was then computed using the following formula:

$$ CC(t)=\frac{\frac{1}{p}\sum \limits_{i=1}^p\left[{a}_x(i){a}_x\left(i+t\right)+{a}_y(i){a}_y\left(i+t\right)+{a}_z(i){a}_z\left(i+t\right)\right]}{{\left\{\frac{1}{p}\sum \limits_{i=1}^p\left[{a}_x{(i)}^2+{a}_y{(i)}^2+{a}_z{(i)}^2\right]\right\}}^{\frac{1}{2}}{\left\{\frac{1}{p}\sum \limits_{i=1}^p\left[{a}_x{\left(i+t\right)}^2+{a}_y{\left(i+t\right)}^2+{a}_z{\left(i+t\right)}^2\right]\right\}}^{\frac{1}{2}}} $$

Where *t* is the time index and *p* is the length of the template wave. If the acceleration change is caused by gait motion, the *CC*(*t*) peaks exhibit alternate changes in the magnitude with time owing to left/right body sway during walking [6, 14, 15, 17].

#### Data analysis

Gait cycle duration and acceleration amplitude were measured from the gait-induced acceleration signals. The gait cycle was defined as the time between successive same foot contact with the ground. Since gait accelerations correlate with ground reaction forces, gait acceleration amplitude was used as an index of ground reaction forces. The duration of the gait cycle and amplitude of gait-related accelerations were averaged for each 10-min recording. The mean gait cycle duration and gait acceleration for each subject were calculated for the entire 24-h period [6, 14, 15, 17].

### Statistical analysis

All normally distributed clinical parameters (e.g., age, disease duration, and MMSE) are expressed as mean ± standard deviation. The Student’s *t*-test was used to compare the clinical parameters of the PD patients and control subjects. The relationships of the mean gait cycle duration and the mean gait acceleration amplitude in PD patients with the disease duration, MMSE score, UPDRS Part III score, and PIGD score were examined using multiple linear regression analysis after adjustment for age, sex, and height. In addition, the relationship between the PIGD score and the MMSE score was examined in a similar manner. A *p* value of *<* 0.05 was considered statistically significant. All statistical analyses were performed using IBM SPSS Statistics (version 22.0, IBM Corp, Armonk, NY).

## Results

We studied 106 consecutive PD patients (62 males and 44 females, mean age: 68.0 ± 6.7 years). Table 1 summarizes the clinical features of PD patients. The mean gait cycle duration in PD patients was 1.18 ± 0.12 s, which was not significantly different from that of the normal control subjects (1.15 ± 0.12 s). The mean gait cycle duration in PD patients correlated significantly with the PIGD score (*β =* − 0.316, *p =* 0.001), but not with the disease duration, MMSE score, or UPDRS Part III score (Table 2) (Additional file 2). On the other hand, the mean gait acceleration amplitude in PD patients was 1.83 ± 0.36 m/sec^2^, which was significantly lower than that of the normal control subjects (2.48 ± 0.60 m/sec^2^, *p <* 0.001). The mean gait acceleration amplitude in PD patients correlated significantly with the MMSE score (*β =* 0.197, *p =* 0.028), UPDRS Part III score (*β =* − 0.327, *p <* 0.001), and PIGD score (*β =* − 0.235, *p =* 0.008), but not with disease duration (Table 2) (Additional file 2). The relationship between the mean gait acceleration amplitude and the MMSE score remained significant even after adding the PIGD score as an adjustment factor (*β =* 0.197, *p =* 0.024). On the other hand, there was no significant relationship between the PIGD score and the MMSE score (*β* = − 0.002, *p =* 0.985).

**Table 1:** Clinical features of patients with Parkinson’s disease (*n* = 106)

Age (years)	68.0 ± 6.7
Male/female (n)	62/44
Disease duration (years)	2.3 ± 2.6
LEDD	100.8 ± 155.1
MMSE	27.8 ± 2.0
Modified HY stage	2.3 ± 0.6
UPDRS Part III score	17.3 ± 9.0
Gait cycle duration (sec)	1.18 ± 0.12
Gait acceleration amplitude (m/sec^2^)	1.83 ± 0.36

Data are mean ± standard deviation

*LEDD* levodopa equivalent daily dose; *MMSE* Mini-Mental State Examination; *Modified HY stage* modified Hoehn and Yahr stage; *UPDRS Part III score* Unified Parkinson’s disease rating scale part III score

**Table 2:** Relationship of the mean gait cycle duration and the mean gait acceleration amplitude with each parameter

	Mean gait cycle duration			Mean gait acceleration amplitude		
	*β*	95% CI	*p*-value	*β*	95% CI	*p*-value
Disease duration	−0.100	−0.350-0.125	0.340	0.095	−0.009-0.278	0.299
MMSE	−0.049	−0.229-0.147	0.631	0.197	0.023–0.371	0.028
UPDRS III score	−0.004	−0.230-0.230	0.968	−0.327	− 0.503--0.176	< 0.001
PIGD score	−0.316	− 0.514--0.119	0.001	− 0.235	− 0.406--0.064	0.008

Data are presented as standardized regression coefficients (β weights)

*MMSE* Mini-Mental State Examination; *UPDRS Part III score* Unified Parkinson’s disease rating scale part III score; Postural instability and gait disorder (PIGD) score: the sum of UPDRS “PIGD items” 13–15, 29, and 30

## Discussion

Proper assessment of gait disorders in PD patients is not always easy. Since these patients can consciously correct their posture and gait and walk carefully in the front of the examining doctor, the results of such assessments conducted in the examination room sometimes do not reflect the true gait pattern. Thus, for proper assessment of gait disturbances of PD patients, it is important to examine the gait during routine daily activities. Based on this concept, the use of wearable sensors for the assessment of gait disturbance has attracted attention in recent years [4, 5].

The core manifestation of gait disorders in patients with PD is bradykinetic/hypokinetic gait, which is characterized by reduced stride length and walking speed and increased double support time. In contrast, the cadence in PD patients is maintained at levels similar to those of healthy individuals [8, 16, 18–20]. Morris et al. [18] concluded that the fundamental deficit in gait hypokinesia in PD patients is based on impaired internal regulation of stride length, and therefore, their walking speed is adjusted by compensatory regulation of cadence. In this study, no significant difference was found in the mean gait cycle duration between PD patients and the normal controls, suggesting preservation of the rhythm of walking in PD. This finding is consistent with that of previous report [8, 16, 18–20]. However, the mean gait cycle duration varied among PD patients with PIGD score of ≥5, and the overall duration tended to decrease as the PIGD score increased, which led to a negative correlation between the two variables (Additional file 2). We believe that this type of relationship is due to compensatory regulation of cadence as a result of the impaired internal regulation of stride length, which in turn develops with the progression of gait disturbance.

The gait acceleration amplitude is a novel gait parameter that can be computed with the tri-axial accelerometer. A study using a force plate system showed that gait acceleration amplitude correlated with floor reaction forces [21]. Previous studies also described a lower gait acceleration amplitude in patients with early-stage PD, compared with normal subjects [8]. In the present study of patients with relatively early-stage PD (mean disease duration, 2.3 years), the mean gait acceleration amplitude was significantly lower than that of the normal control subjects. Furthermore, the amplitude correlated significantly with the motor symptoms. Furthermore, the mean gait acceleration amplitude of our patients correlated with global cognitive function, independent of the PIGD score. Previous studies on the relationship between gait disturbance and cognitive function in PD showed that gait disturbance was associated with cognitive domains, such as global cognitive function, executive function, attention, visuospatial abilities, and memory [22, 23]. For example, using three-dimensional motion analysis, Kim et al. [24] found a close relation between MMSE score and short-stepped gait in their PD patients with a mean disease duration of 4.8 years. In addition, another study of patients with advanced-stage PD demonstrated the association of PIGD score with global cognitive function, as evaluated by the Montreal Cognitive Assessment and the Mattis Dementia Rating Scale-2. Moreover, among patients with advanced-stage PD, the prevalence of dementia was higher in patients with the PIGD subtype than in those with the tremor dominant (TD) subtype. Furthermore, the PIGD subtype was described as a significant predictor of PD-associated dementia [25, 26]. However, in patients with early-stage PD, no clear differences in cognitive function have been established between the PIGD and TD subtypes [27, 28]. The present study showed a significant association between the mean gait acceleration amplitude and MMSE score although the subjects had relatively early-stage PD with MMSE scores of ≥20. The results showed that gait disturbance was associated with overall cognitive function even in patients with relatively early-stage PD. The results also suggested that gait analysis under free-living conditions may be useful in the prediction of cognitive function.

In the present study, the gait of PD patients was monitored and analyzed using the MIMAMORI-Gait wearable sensor. The daily physical activities of PD patients included involuntary and voluntary adjustment of posture and gait according to changes in the surrounding environment and walking conditions. In this regard, walking under free-living conditions requires more advanced control than walking in the examination room during clinical checkup. The mean gait acceleration amplitude, computed from the MIMAMORI-Gait recording, reflected the gait characteristics described in various motor-related diseases, including cognitive function disorders [8–11], and was therefore useful in the assessment of the gait disturbance in PD patients. Further studies are needed to determine the effects of treatment (e.g., levodopa) on various gait parameters as well as cognitive function, and the feasible use of the MIMAMORI-Gait device to predict gait freeze and falls based on gait analysis under free-living conditions.

The present study has several limitations. First, the study participants were at a relatively early stage of the disease (mean duration, 2.3 years) because the study enrolled patients with PD free of motor complications. Furthermore, patients with moderate and severe cognitive impairment, defined by a MMSE score of < 20, were excluded because such patients were expected to have difficulty in wearing the device according to the predefined procedure as well as in completing continuous recording using the MIMAMORI-Gait sensor. After obtaining the measurements, we confirmed that the enrolled patients wore the device as instructed and their gait was continuously measured. When using the MIMAMORI-Gait sensor, it may be more preferable, under ordinary circumstances, to measure the gait parameters several times and to use the mean values for the parameters due to differences in engagement in daily activities among the patients. To check for this, we assessed the data obtained from one measurement session in the present study. To complement this, the recordings were conducted on a day that did not include any special events, such as no travels, and the patients were instructed beforehand to spend the day as usual. To assess cognitive function, MMSE was used in the present study. Although impairments of various cognitive domains, such as the executive function, attention and working memory, language, memory, and visuospatial function, have been described in the early stages of PD [29], these domains were not analyzed in the present study. Further studies that include patients with advanced-stage PD are needed to investigate the relationship between each cognitive domain and gait parameters.

## Conclusion

Analysis of gait data recorded non-invasively over the 24-h under free-living conditions showed preservation of the gait rhythm in PD patients at levels similar to those of the normal subjects. However, the mean gait acceleration amplitude was significantly reduced in PD patients relative to the control. Moreover, the mean gait acceleration amplitude correlated with the severity of motor symptoms and global cognitive function. We believe that a comprehensive assessment of gait disturbance in patients with PD should take into consideration the cognitive function status of the patients.

## Supplementary information

**Additional file 1.** Results of measurements in a 77-year-old man with Parkinson’s disease: (a) The ordinate represents the gait cycle duration, and the abscissa represents time. (b) The ordinate represents the acceleration of all movements including gait, and the abscissa represents time. Note the decreased in acceleration amplitude during sleep (time from 23:30 to 06:00.

**Additional file 2.** (a) Relationship between mean gait cycle duration and PIGD score, (b) Relationship between mean gait acceleration amplitude and PIGD score.

## Data Availability

The datasets used and/or analyzed during the study are available from the corresponding author on reasonable request.

## Abbreviations

PD: Parkinson’s disease
MMSE: Mini-Mental State Examination
UPDRS: Unified Parkinson’s Disease Rating Scale
PIGD: Postural instability and gait disorder
MDS-UPDRS: Movement Disorder Society-sponsored version of the UPDRS
HY: Hoehn and Yahr

## References

[1] Yamawaki M, Kusumi M, Kowa H, Nakashima K (2009). Changes in prevalence and incidence of Parkinson's disease in Japan during a quarter of a century. Neuroepidemiology.

[2] Mirelman A, Bonato P, Camicioli R, Ellis TD, Giladi N, Hamilton JL (2019). Gait impairments in Parkinson's disease. Lancet Neurol.

[3] Goetz CG, Tilley BC, Shaftman SR, Stebbins GT, Fahn S, Martinez-Martin P (2008). Movement Disorder Society UPDRS revision task force. Movement Disorder Society-sponsored revision of the unified Parkinson's disease rating scale (MDS-UPDRS): scale presentation and clinimetric testing results. Mov Disord.

[4] Del Din S, Godfrey A, Mazzà C, Lord S, Rochester L (2016). Free-living monitoring of Parkinson's disease: lessons from the field. Mov Disord.

[5] Rovini E, Maremmani C, Cavallo F (2017). How wearable sensors can support Parkinson's disease diagnosis and treatment: a systematic review. Front Neurosci.

[6] Mitoma H, Yoneyama M, Orimo S (2010). 24-hour recording of parkinsonian gait using a portable gait rhythmogram. Intern Med.

[7] Utsumi H, Terashi H, Ishimura Y, Takazawa T, Hayashi A, Mochizuki H (2012). Quantitative assessment of gait bradykinesia in Parkinson's disease using a portable gait rhythmogram. Acta Med Okayama.

[8] Terashi H, Utsumi H, Ishimura Y, Mitoma H (2013). Independent regulation of the cycle and acceleration in parkinsonian gait analyzed by a long-term daily monitoring system. Eur Neurol.

[9] Iijima M, Mitoma H, Uchiyama S, Kitagawa K (2017). Long-term monitoring gait analysis using a wearable device in daily lives of patients with Parkinson's disease: the efficacy of Selegiline hydrochloride for gait disturbance. Front Neurol.

[10] Hatanaka N, Sato K, Hishikawa N, Takemoto M, Ohta Y, Yamashita T (2016). Comparative gait analysis in progressive supranuclear palsy and Parkinson's disease. Eur Neurol.

[11] Terashi H, Utsumi H, Ishimura Y, Aizawa H, Yoneyama M, Mitoma H (2015). Kinematic analysis of 24-hour recording of walking pattern in patients with vascular parkinsonism. Int J Neurosci.

[12] Gibb WR, Lees AJ (1988). The relevance of the Lewy body to the pathogenesis of idiopathic Parkinson's disease. J Neurol Neurosurg Psychiatry.

[13] Jankovic J, McDermott M, Carter J, Gauthier S, Goetz C, Golbe L (1990). Parkinson study group. Variable expression of Parkinson's disease: a base-line analysis of the DATATOP cohort. Neurology.

[14] Yoneyama M, Kurihara Y, Watanabe K, Mitoma H (2013). Accelerometry-based gait analysis and its application to Parkinson's disease assessment- part 2: a new measure for quantifying walking behavior. IEEE Trans Neural Syst Rehabil Eng.

[15] Yoneyama M, Mitoma H, Okuma Y (2013). Accelerometry-based long-term monitoring of movement disorders: from diurnal gait behavior to nocturnal bed mobility. J Mech Med Biol.

[16] Giladi N, Horak FB, Hausdorff JM (2013). Classification of gait disturbances: distinguishing between continuous and episodic changes. Mov Disord.

[17] Yoneyama M, Kurihara Y, Watanabe K, Mitoma H (2014). Accelerometry-based gait analysis and its application to Parkinson's disease assessment- part 1: detection of stride event. IEEE Trans Neural Syst Rehabil Eng.

[18] Morris ME, Iansek R, Matyas TA, Summers JJ (1994). The pathogenesis of gait hypokinesia in Parkinson's disease. Brain.

[19] Morris M, Iansek R, McGinley J, Matyas T, Huxham F (2005). Three-dimensional gait biomechanics in Parkinson's disease: evidence for a centrally mediated amplitude regulation disorder. Mov Disord.

[20] Svehlík M, Zwick EB, Steinwender G, Linhart WE, Schwingenschuh P, Katschnig P (2009). Gait analysis in patients with Parkinson's disease off dopaminergic therapy. Arch Phys Med Rehabil.

[21] Suzuki M, Yogo M, Morita M, Terashi H, Iijima M, Yoneyama M, Takada M, Utusmi H, Okuma Y, Hayashi A, Orimo S, Mitoma H**.** A proposal for new algorithm that defines gait-induced acceleration and gait cycle in daily parkinsonian gait disorders. In: Wearable Technologies. Ortiz JH, ed. InTech Open, pp25–48. http://dx.doi.org/10.5772/intechopen.75483.

[22] Amboni M, Barone P, Hausdorff JM (2013). Cognitive contributions to gait and falls: evidence and implications. Mov Disord.

[23] Kelly VE, Johnson CO, McGough EL, Shumway-Cook A, Horak FB, Chung KA (2015). Association of cognitive domains with postural instability/gait disturbance in Parkinson's disease. Parkinsonism Relat Disord.

[24] Kim SM, Kim DH, Yang Y, Ha SW, Han JH (2018). Gait patterns in Parkinson's disease with or without cognitive impairment. Dement Neurocogn Disord.

[25] Alves G, Larsen JP, Emre M, Wentzel-Larsen T, Aarsland D (2006). Changes in motor subtype and risk for incident dementia in Parkinson's disease. Mov Disord.

[26] Burn DJ, Rowan EN, Allan LM, Molloy S, O'Brien JT, McKeith IG (2006). Motor subtype and cognitive decline in Parkinson's disease, Parkinson's disease with dementia, and dementia with Lewy bodies. J Neurol Neurosurg Psychiatry.

[27] Domellöf ME, Elgh E, Forsgren L (2011). The relation between cognition and motor dysfunction in drug-naive newly diagnosed patients with Parkinson's disease. Mov Disord.

[28] Domellöf ME, Ekman U, Forsgren L, Elgh E (2015). Cognitive function in the early phase of Parkinson’s disease, a five-year follow-up. Acta Neurol Scand.

[29] Litvan I, Goldman JG, Tröster AI, Schmand BA, Weintraub D, Petersen RC (2012). Diagnostic criteria for mild cognitive impairment in Parkinson's disease: Movement Disorder Society task force guidelines. Mov Disord.

