# Supplementary figures and images for: Analysis of non-invasive gait recording under free-living conditions in patients with Parkinson’s disease: relationship with global cognitive function and motor abnormalities
Source: BMC Neurol. 2020 Apr 29;20:161. doi: 10.1186/s12883-020-01729-w (PMC7189597; doi:10.1186/s12883-020-01729-w)

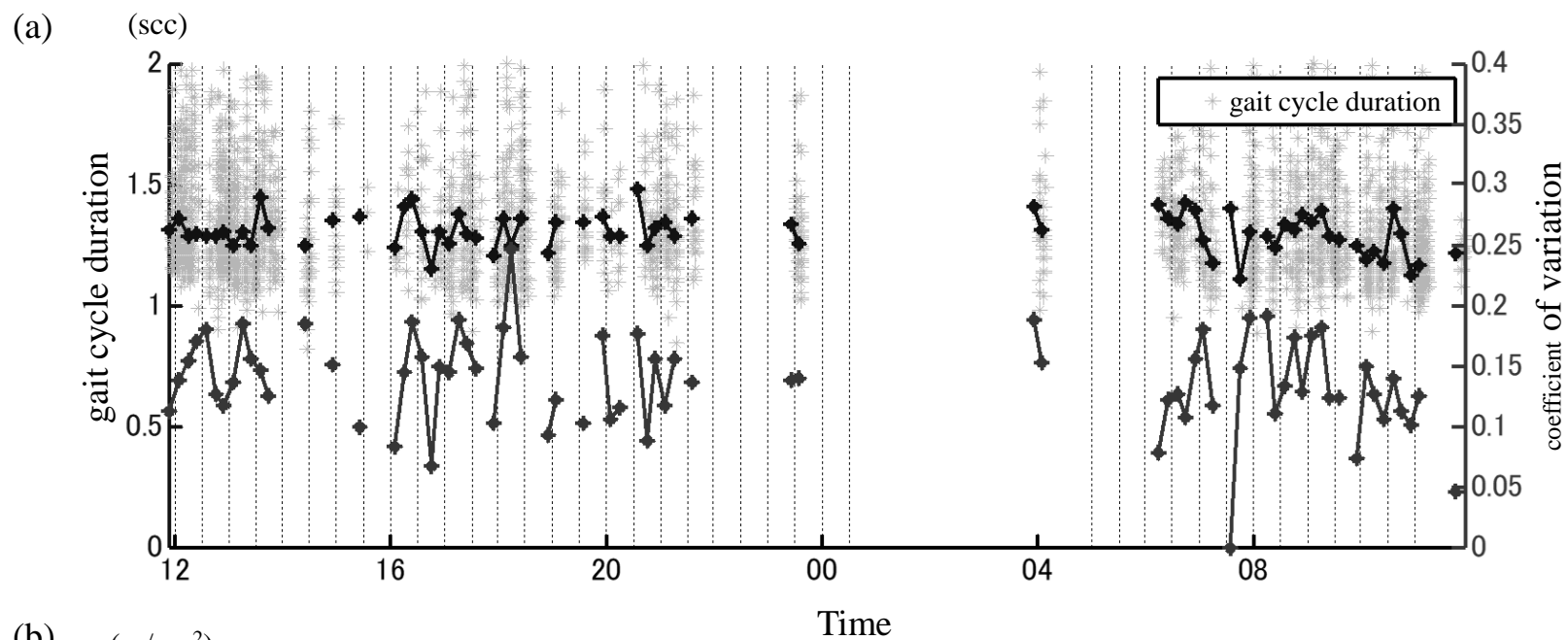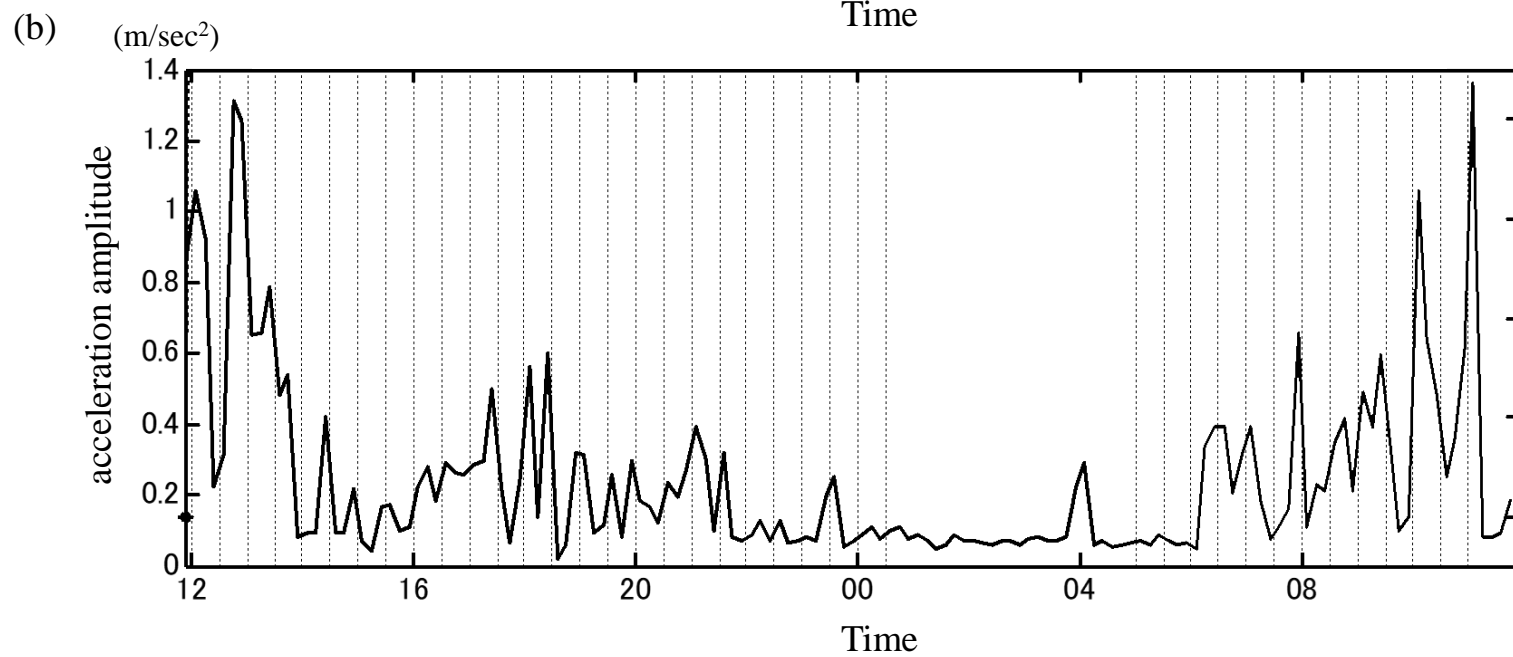

Supplement: Supplementary file 1 — Additional file 1. Results of measurements in a 77-year-old man with Parkinson’s disease: (a) The ordinate represents the gait cycle duration, and the abscissa represents time. (b) The ordinate represents the acceleration of all movements including gait, and the abscissa represents time. Note the decreased in acceleration amplitude during sleep (time from 23:30 to 06:00. [file 12883_2020_1729_MOESM1_ESM.pdf]

(a)

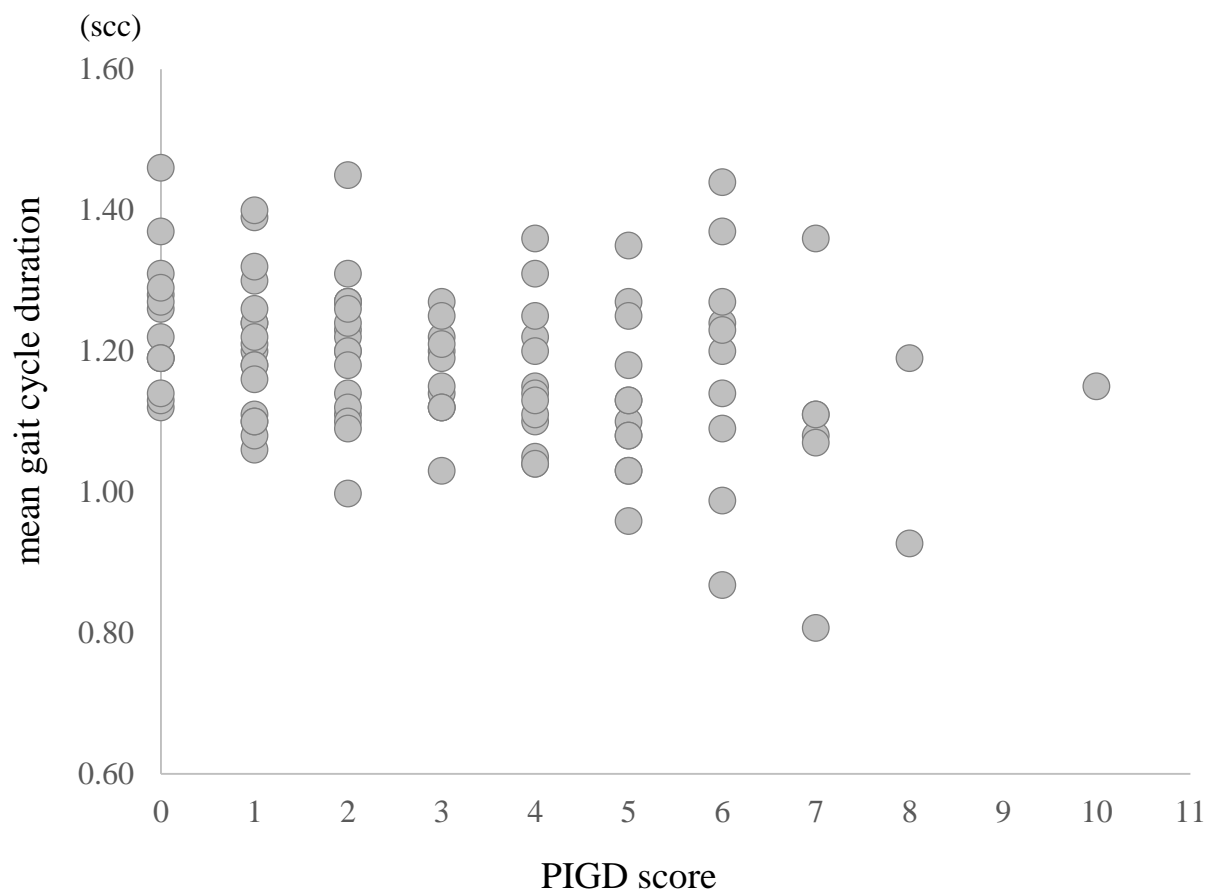

(b)

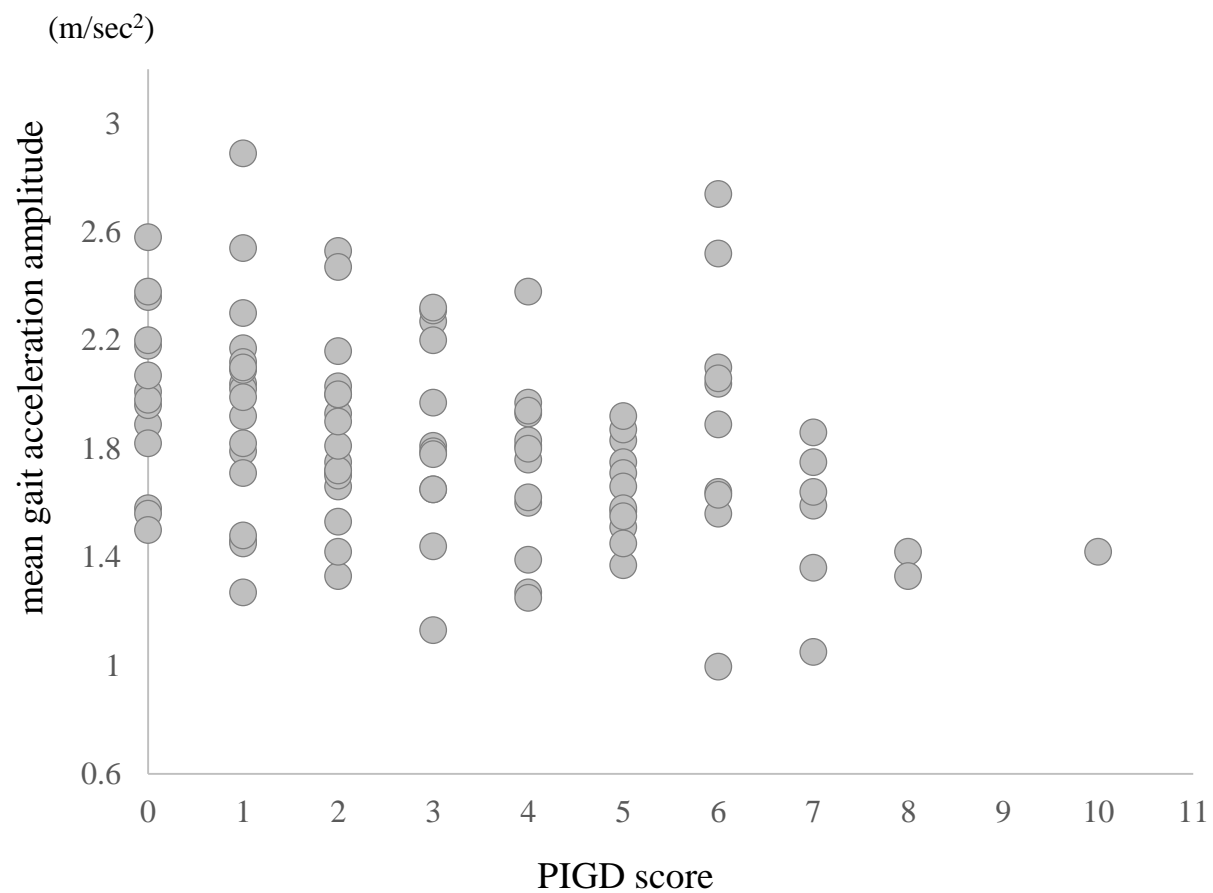

Supplement: Supplementary file 2 — Additional file 2. (a) Relationship between mean gait cycle duration and PIGD score, (b) Relationship between mean gait acceleration amplitude and PIGD score. [file 12883_2020_1729_MOESM2_ESM.pdf]
